# Penicillin Concentrations in Oropharyngeal and Frontal Sinus Tissue Following Intravenous Bolus and Continuous Infusion - An Experimental Porcine Study

**DOI:** 10.1007/s11095-026-04017-3

**Published:** 2026-01-23

**Authors:** Pelle Hanberg, Hans Christian Rasmussen, Mats Bue, Maiken Stilling, Andrea René Jørgensen, Elisabeth Krogsgaard Petersen, Johanne Gade Lilleøre, Magnus A. Hvistendahl, Jesper Bille, Tejs Ehlers Klug

**Affiliations:** 1 Department of Otorhinolaryngology, Head and Neck Surgery, Aarhus University Hospital, Palle Juul-Jensens Boulevard 99, 8200, Aarhus N Aarhus, Denmark; 2 Department of Clinical Medicine, Aarhus University, FORUM, Palle Juul-Jensens Boulevard 11, 8200, Aarhus N Aarhus, Denmark; 3 Aarhus Denmark Microdialysis Research (ADMIRE), Orthopaedic Research Laboratory, Aarhus University Hospital, Aarhus N Aarhus, Denmark; 4 Department of Orthopaedic Surgery, Aarhus University Hospital, Aarhus N Aarhus, Denmark

**Keywords:** antibiotic, continuous infusion, frontal sinus tissue, oropharyngeal tissue, penicillin, pharmacokinetics

## Abstract

**Background and Purpose:**

Studies have documented that continuous infusion is superior to bolus infusion in providing longer time with drug concentration above the minimal inhibitory concentration (T > MIC). This porcine study compared steady-state penicillin concentrations in oropharyngeal and frontal sinus tissues following intravenous bolus and continuous administration.

**Experimental Approach:**

Twelve pigs were randomized to receive either intravenous bolus (Group BI) or continuous (Group CI) infusion of penicillin (1.2 g). Doses were administered at 0, 6, and 12 h, with sampling from 12 to 18 h. Microdialysis was used for sampling in oropharyngeal and frontal sinus tissues, with simultaneous plasma sampling. The primary endpoints were T > MIC for two MIC targets (0.125 (low target) and 0.5 (high target) μg/mL) and attainment of ≥ 50%T > MIC treatment target.

**Key Results:**

No statistically significant differences were found between Group BI and CI for either MIC target. The ≥ 50%T > MIC target was achieved in all compartments except for the high MIC target in oropharyngeal tissue in Group CI (46%). although no statistical significance, T > MIC in oropharyngeal tissue tended to be longer in Group BI (low target: 98%; high target: 74%) compared with Group CI (low target: 68%; high target: 46%) (*p* = 0.07 and *p* = 0.19, respectively).

**Conclusion and Implication:**

Penicillin bolus and continuous infusion resulted in comparable T > MIC in oropharyngeal and frontal sinus tissues. However, bolus infusion showed a higher likelihood of attaining ≥ 50%T > MIC in oropharyngeal tissue. These findings are specific to the porcine model and dosing regimens used and cannot be directly extrapolated to humans.

## Introduction

In Northern European countries, penicillin is the preferred antibiotic for the treatment of oropharyngeal and paranasal sinus infections [1, 2], as the prevalent bacterial pathogens involved in oropharyngeal infections are Group A Streptococci (GAS) and *Fusobacterium necrophorum* (FN), while *Streptococcus pneumoniae* (SP) and *Haemophilus influenzae* (HI) are the most common causes of acute sinusitis [2, 3].

Penicillin’s antibacterial effectiveness is best related to the time with free drug concentrations above the minimal inhibitory concentration of the specific pathogen (T > MIC) [4]. Optimal bactericidal penicillin activity has been described when achieving T > MIC in at least half of the treatment time (≥ 50%T > MIC) [4]. Most GAS, FN, SP, and HI isolates exhibit MICs lower than 0.125 μg/mL, though some isolates exhibit MICs as high as 0.5–1 μg/mL [5, 6].

Penicillin comes in different administration formulations, allowing for an uncomplicated switch between administration forms (enteral and intravenous) regarding bacterial coverage. In a recent study, we compared oropharyngeal and frontal sinus tissue concentrations of penicillin following enteral and intravenous bolus administration, finding significantly higher tissue T > MIC following intravenous administrations [7]. When penicillin was administered enterally, neither oropharyngeal nor frontal sinus tissue reached the treatment target of 50%T > MIC. Following intravenous administrations, the low MIC target (0.125 μg/mL) was reached in both tissues, while only oropharyngeal tissue reached the high MIC target (0.5 μg/mL).

Multiple studies have documented that continuous infusion is superior to bolus infusion in providing longer T > MIC [8–11]. Further stressing the clinical potential of continuous infusion, a recent study reported a decreased mortality among critically ill patients when beta-lactam antibiotics, not including penicillin, were administered as prolonged infusion (including continuous infusion) compared to bolus infusion [12]. Lately, beta-lactam antibiotics, including penicillin, have been offered as outpatient parenteral antimicrobial therapy (OPAT) for which the antibiotics were administered as continuous infusion [13, 14]. However, studies comparing penicillin target tissue concentrations following continuous and bolus infusion are absent in the literature.

This study aimed to determine and compare the oropharyngeal and frontal sinus tissue concentrations of penicillin following continuous and bolus infusion in a steady-state treatment setting using a porcine model. The primary endpoints were T > MIC and attainment of the treatment target of ≥ 50%T > MIC for 0.125 μg/mL (low MIC target) and 0.5 μg/mL (high MIC target).

## Material and Methods

This study was approved by the Danish Animal Experiments Inspectorate (license number: 2021–15–0201–01094) and was carried out at the facilities of the Department of Clinical Medicine, Aarhus University. The study was performed in agreement with national and international regulations and designed in accordance with the ARRIVE guidelines. To comply with the 3Rs and the mandatory reduction of the needed number of animals, the animals used in the bolus infusion group have provided data for two other studies [7, 15]. None of the data presented in this study has been published elsewhere.

### Study Design

Twelve pigs (crossbreed of Danish landrace, Danish Duroc, and Danish Yorkshire) (weight: 68–78 kg), were included. The pigs were randomized in pairs of two to receive 1.2 g of intravenous penicillin as either bolus infusion (Group BI) or as continuous infusion (Group CI). Penicillin was administered every six hours, and dialysate and plasma samples were collected during the third dosing interval, simulating a steady-state scenario.

### Microdialysis

Microdialysis is a pharmacokinetic tool that allows for dynamic sampling of molecules of interest from multiple target tissues, simultaneously [16, 17]. In the case of antibiotics, only the unbound and, thereby, pharmaceutical active fractions are sampled [18]. To determine the absolute concentration, catheter calibration is imperative [16, 17]. In this study, retrodialysis by drug was used with Penicillin V as an internal calibrator for Penicillin G, employing a solution of Penicillin V (0.9% NaCl containing 5 µl/mL). Elaboration of the microdialysis and calibration technique can be found elsewhere [7].

The study utilized microdialysis equipment consisting of the 63 microdialysis catheter (with a membrane length of either 10 mm or 30 mm and a molecular cut-off of 20 kDa), the 107 microdialysis pump, and splitable introducers, all supplied by M Dialysis AB, Stockholm, Sweden. The pump was set at a perfusion rate of 2 μl/min.

### Study Procedures

Throughout the study, the pigs were kept under general anesthesia using a combination of propofol (10 mg/mL, 400–650 mg/h, B. Braun Medical, Melsungen, Germany) and fentanyl (50 μg/mL, 0.6–2.4 mg/h, Hameln Pharma GmbH, Germany). The temperature was maintained between 36.1°C and 39.2°C, while the pH was kept within a range of 7.4 to 7.6. Continuous monitoring of vital parameters was performed, with adjustments made as needed.

After initiated anesthesia, the right frontal sinus was accessed externally using a 10 mm drill. A catheter with a 10 mm membrane was inserted into the sinus cavity, and the entry site was sealed with bone wax. Using a splitable introducer, another catheter with a 30 mm membrane was placed in the submucosal tissue of the oropharyngeal region, superior and medial to the right palatine tonsil. Both catheters were secured with a single suture in adjacent tissues. Following catheter placement, the catheters were perfused with 0.9% NaCl containing 5 μg/mL Penicillin V for continuous calibration.

The pigs were randomized (in blocks of two) to one of two groups: Group BI (*n* = 6) and Group CI (*n* = 6), both receiving 1.2 g of Penicillin G (Benzylpenicillin Panpharma 1.2 g, powder for injection/infusion, batch 307,062, Panpharma, Luitré, France). Group BI received the penicillin dose over 20 min, while Group CI received the penicillin dose continuously during the 6-h dosing interval. Penicillin was administered at time 0, 6, and 12 h, with the start of the first penicillin infusion defining time 0. Prior to the first continuous dosing in Group CI, an initial bolus dose (1.2 g) was administered to resemble standard clinical practice. As such, the total administered dose amounted to 3.6 g in Group BI and 4.8 g in Group CI.

Sampling was conducted from time 12 to 18 h. A baseline sample was collected before the third dosing interval was initiated and collected at 12 h. Dialysate samples were collected at 15-min intervals from time 12 to 13 h, at 30-min intervals from time 13 to 15 h, and at 60-min intervals from time 15 to 18 h, resulting in a total of 12 dialysates from both tissues. Blood samples were drawn from a central venous catheter at the midpoint of each dialysate sampling interval. Blood samples were centrifuged at 2,000 g for 10 min at 5°C. Both plasma aliquots and dialysates were stored at −80°C until further analysis.

### Quantification of penicillin concentrations

Plasma and dialysate concentrations were determined using an Exion-AD HPLC and Sciex 4500 qTrap MS system. For both Penicillin G and Penicillin V, the limit of quantification was 0.05 μg/mL. The CV percentage was below 10, calculated at concentrations of 0.05, 0.5, and 1 mg/L. Dialysate concentrations are given as free concentrations, while plasma concentrations are given as total concentrations [7]. The quantification technique of Penicillin G and V has been elaborated elsewhere [7].

### Pharmacokinetic analysis and statistics

The estimation of T > MIC was performed using linear interpolation in Microsoft Excel (version 16.16.11, Microsoft Corporation, Redmond, Washington) for time 12 to 18 h. Pharmacokinetic parameters for each compartment across all pigs were assessed using non-compartmental analysis in Stata (version 15.1, StataCorp, College Station, TX, United States). The areas under the concentration–time curves (AUC_12–18 h_) were calculated using the linear trapezoidal rule. The highest observed concentration among all measurements was defined as the peak drug concentration (C_max_), which enabled the determination of the time to reach C_max_ (T_max_). The half-life (T_1/2_) was calculated using the formula ln[2]/λeq, where λeq is the terminal elimination rate constant obtained from linear regression of the log-transformed concentration over time. T_max_ and T_1/2_ were only calculated for Group BI. Due to the small sample size, the Kenward-Roger approximation was applied for degrees of freedom adjustment. A linear mixed model with repeated measures analysis of variance (ANOVA) was used to analyze and compare T > MIC and pharmacokinetic parameters, accounting for interactions between groups and compartments. Pairwise comparisons were conducted using linear combination. The normality and homogeneity of variance of residuals were evaluated through visual inspection of residuals, fitted values, and random effects estimates. As these assumptions were found to be violated for the pharmacokinetic parameters, log transformation was applied before statistical analysis. The T > MIC data are presented as means with 95% confidence intervals, while the pharmacokinetic data are presented as medians with 95% confidence intervals. A significance level of 5% was used for all statistical tests. Penicillin concentrations in the dialysate were assigned to the midpoint of the sampling intervals before analysis.

## Results

All twelve pigs completed the study, and all catheters functioned throughout the study period. The mean relative recovery ranged from 0.38–0.58 (SD range: 0.02–0.23).

### T > MIC 0.125 (low target) and 0.5 μg/mL (high target)

**Fig. 1:**
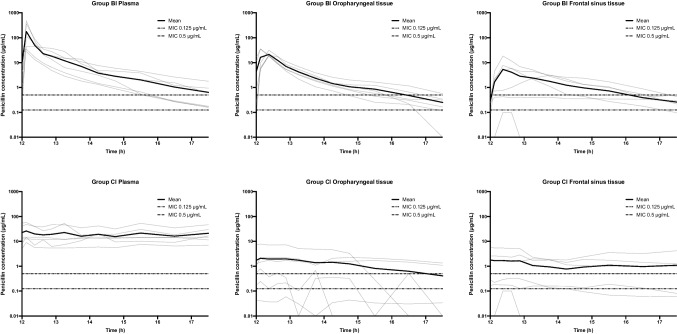
Individual (gray lines) and mean (bold lines) concentration–time profiles for penicillin in plasma (total concentration), oropharyngeal (free concentration), and frontal sinus tissue (free concentration) following intravenous bolus (Group BI) and continuous infusion (Group CI). The dotted line represents minimal inhibitory concentration (MIC) of 0.125 and 0.5 μg/mL. The y-axis is in log scale.

The mean T > MIC (0.125 and 0.5 μg/mL) are presented in Table I and II, and the concentration–time profiles are presented in Fig. 1. Though no statistically significant differences were found between Group BI and CI for both the low and high MIC targets, T > MIC for oropharyngeal tissue tended to be longer for both MIC targets in Group BI (low target: 98% and high target: 74%) compared to Group CI (low target: 68% and high target: 46%) (low target: *p* = 0.07 and high target: *p* = 0.19).

**Table I:** Time with Penicillin Concentrations Above the Minimum Inhibitory Concentration (T > MIC) in Plasma (total Concentration), Oropharyngeal (free Concentration), and Frontal Sinus Tissue (free Concentration) Stratified By Administration Form

	**Percentage of time with T > MIC (95%CI)**		**Absolute time with T > MIC (95%CI)**		
	Group		Group		Comparison: Group BI vs Group CI
	BI	CI	BI	CI	
	**Low MIC target**		**Low MIC Target**		*p*
Plasma	100% (76–124)	100% (76–124)	330 min (250–410)	330 min (250–410)	1.00
Oropharyngeal tissue	98% (74–122)	68% (44–92)	323 min (243–403)	224 min (144–304)	0.07
Frontal sinus tissue	82% (58–106)	71% (47–95)	271 min (192–351)	234 min (154–314)	0.50
	**High MIC target**		**High MIC target**		
Plasma	79% (49–104)	100% (70–130)	261 min (162–342)	330 min (231–429)	0.31
Oropharyngeal tissue	74% (44–104)	46% (17–76)	244 min (145–342)	153 min (55–252)	0.19
Frontal sinus tissue	51% (21–80)	50% (20–80)	167 min (68–265)	165 min (66–264)	0.98

*BI*: bolus infusion; *CI*: continuous infusion; *n*: number; *MIC*: minimal inhibitory concentration; *min*: minutes; *Low MIC target*: 0.125 μg/mL; *High MIC target*: 0.5 μg/mL

*P* values were calculated using linear combinations

**Table II:** Comparisons of T > MIC Between Plasma, Oropharyngeal Tissue, and Frontal Sinus Tissue Within Group BI and CI, Respectively

Group	MIC Target	Plasma vs oropharyngeal tissue	Plasma *vs* frontal sinus tissue	Oropharyngeal tissue *vs* frontal sinus tissue
BI	Low	0.90	0.29	0.35
	High	0.80	0.17	0.26
CI	Low	0.055	0.08	0.86
	High	**0.010**	**0.016**	0.86

*T > MIC*: Time with penicillin concentrations above the minimum inhibitory concentration; *BI* bolus infusion; *CI* continuous infusion; *Low MIC target*: 0.125 μg/mL; *High MIC target*: 0.5 μg/mL

**Bold** highlights statistically significant differences

For comparison of the high MIC target within groups, T > MIC was significantly longer in plasma compared to oropharyngeal and frontal sinus tissue in Group CI.

The treatment target of ≥ 50%T > MIC was reached in both groups in all compartments except for the high MIC target in oropharyngeal tissue in Group CI (46%).

### Pharmacokinetics

The pharmacokinetic parameters are given in Table III and IV. C_max_ was significantly higher in oropharyngeal tissue (median: 25.7 μg/mL) in Group BI compared to Group CI (median: 1.0 μg/mL) (*p* = 0.003). No other statistically significant differences were found regarding AUC and C_max_ between groups.

**Table III:** Pharmacokinetic Parameters for Penicillin Following Intravenous Bolus Infusion (Group BI) and Continuous Infusion (Group CI) in Plasma (total concentration), Oropharyngeal (free concentration), and Frontal Sinus Tissue (free concentration)

Parameter	Group BI	Group CI	Comparison: Group BI vs Group CI
**AUC**_**12–18 h**_** (min μg/mL)**			*p*
Plasma	3,320 (643–17,131)	5,202 (1,008–26,847)	0.70
Oropharyngeal tissue	1,146 (222–5,915)	151 (29–778)	0.08
Frontal sinus tissue	136 (26–701)	76 (15–393)	0.61
**C**_**max**_** (μg/mL)**			
Plasma	98.7 (22.9–426.3)	22.4 (5.2–96.5)	0.15
Oropharyngeal tissue	25.7 (5.9–110.8)	1.0 (0.2–4.3)	0.003
Frontal sinus tissue	1.5 (0.3–6.4)	0.6 (0.1–2.6)	0.37
**T**_**max**_** (min)**			
Plasma	9 (4–23)	-	-
Oropharyngeal tissue	16 (7–35)	-	-
Frontal sinus tissue	34 (15–77)	-	-
**T**_**1/2**_** (min)**			
Plasma	56 (25–124)	-	-
Oropharyngeal tissue	65 (29–145)	-	-
Frontal sinus tissue	277 (114–673)	-	-

Values are given as medians (95%CI)

*AUC*: area under the concentration time curve; *min*: minutes; *C*_max_: peak drug concentration; *T*_max_: time to C_max_; *T*_1/2_: half-life

*P* values were calculated using linear combinations

**Table IV:** Comparisons of Pharmacokinetic Parameters Between Plasma, Oropharyngeal Tissue, and Frontal Sinus Tissue Within Group BI and CI, Respectively

Parameter	Group	Plasma vs oropharyngeal tissue	Plasma vs frontal sinus tissue	Oropharyngeal vs frontal sinus tissue
AUC	BI	0.36	**0.01**	0.08
	CI	**0.005**	**0.001**	0.55
C_max_	BI	0.20	**0.001**	**0.01**
	CI	**0.006**	**0.002**	0.61
T_max_	BI	0.40	**0.04**	0.17
T_1/2_	BI	0.76	**0.01**	**0.02**

*BI*: bolus infusion; *CI*: continuous infusion; *AUC*: area under the concentration time curve; *C*_max_: peak drug concentration; *T*_max_: time to *C*_max_; *T*_1/2_: half-life

**Bold** highlights statistically significant differences

## Discussion

To the best of our knowledge, this is the first study to explore and compare penicillin tissue concentrations in oropharyngeal and frontal sinus tissues following intravenous bolus and continuous administration. We found no significant differences in oropharyngeal and frontal sinus tissue concentrations when comparing bolus with continuous infusion, but T > MIC tended to be longer when penicillin was administered as bolus infusion.

Multiple studies on beta-lactam antibiotics have previously found continuous infusion superior to bolus infusion when evaluating the T > MIC, which has favored continuous infusion at the intensive care units [8–12, 19, 20]. This study suggests that intravenous bolus infusion of penicillin is non-inferior to continuous infusion concerning T > MIC in oropharyngeal and frontal sinus tissues. Notably, the oropharyngeal tissue T > MIC, for both the low (98% vs 68%) and high (74% vs 46%) MIC targets, tended to be longer in Group BI compared to Group CI. Furthermore, fewer pigs in Group CI reached the treatment target of ≥ 50%T > MIC for both the low and high MIC target in oropharyngeal tissue compared to Group BI (Group CI: 4 of 6 pigs for the low target and 3 of 6 pigs for the high target; Group BI: 6 of 6 pigs for the low target and 5 of 6 pigs for the high target).

A distinctive feature of Group CI for both oropharyngeal and frontal sinus tissues was the tendency to achieve either very high (nearly 100%) or very low (approaching 0%) T > MIC, which emphasizes the importance of sufficient tissue penetration to prevent subtherapeutic concentrations throughout the dosing interval when penicillin is administered as continuous infusion. This study suggests that the risk of subtherapeutic tissue concentrations can be decreased substantially using penicillin bolus infusion. Alternatively, higher loading and/or daily doses should be considered when penicillin is administered as continuous infusion.

A possible explanation for the apparently increased tissue exposure following bolus infusion, despite comparable plasma concentrations between regimens, may be the transiently higher peak plasma concentrations (C_max_) obtained after bolus dosing. The resulting steeper plasma–tissue concentration gradient could enhance passive diffusion and facilitate drug penetration into the tissues [21, 22]. In contrast, the relatively constant plasma concentration during continuous infusion may reduce the driving force for diffusion and thereby limit tissue exposure [21, 22]. However, this interpretation remains speculative and warrants further investigation.

With the ambition to reduce healthcare costs and the number of hospital beds, OPAT has gained great popularity for treating various infectious diseases [23]. In OPAT, antibiotics can be administered as both bolus and continuous infusion. Yet, for beta-lactam antibiotics, continuous infusion is preferred to reduce dosing frequency and potentially enhance treatment outcomes [13, 14]. Only a few studies have investigated the use of penicillin for OPAT [13, 24, 25]. These studies found OPAT (using continuous infusion) clinically effective in the cases of erysipelas, endocarditis, and different deep-seated infections. However, based on the results of the current study, continuous infusion of penicillin may be associated with an increased risk of subtherapeutic tissue concentrations, illustrated by shorter T > MIC in oropharyngeal tissue after continuous infusion. This accentuates the importance of conducting pharmacokinetic studies measuring relevant target tissue concentration of antibiotics which may be used in OPAT [26].

When comparing oropharyngeal and frontal sinus tissue T > MIC of penicillin following intravenous bolus administration in this study with our previous findings (investigating penicillin T > MIC during the first dosing interval following intravenous bolus administration), similar T > MIC were observed in oropharyngeal tissue, while higher T > MIC was found in frontal sinus tissue in the present study despite qual dosing [7]. The results of present study are useful for evaluating guidelines for treatment of infections, whereas our previous study better correlates to the clinical situation of prophylactic antibiotic administration. Nevertheless, these results illustrate that prophylactic concentrations can not necessarily be correlated to steady-state concentrations.

One major limitation of this study is the use of anesthetized juvenile pigs (aged 5 months), with potential anesthesia-induced physiological changes and better renal clearance compared to adult patients [27, 28]. Furthermore, our study is based on a rather small population with 6 pigs in each group. Despite the pronounced differences in the T > MIC for oropharyngeal tissue, the relatively small sample size may be accountable for the statistically non-significant results due to high interindividual variability. The small number of animals may also explain the minor deviation observed between plasma and oropharyngeal tissue concentrations during continuous infusion, rather than indicating a true difference in distribution kinetics. To mitigate some of the limitations related to sample size, we used a relatively homogeneous animal model and a pharmacological tool (microdialysis), providing rich tissue individual data for comparison between tissues. Moreover, study only allowed the quantification of total penicillin concentrations in plasma, while free drug concentrations were quantified in oropharyngeal and frontal sinus tissues, being the pharmaceutical active fraction of the drug. Also, the microdialysis catheter was placed in adherence to the mucosa within the lumen of the frontal sinus and not directly within the mucosa. Moreover, it is important to apprehend that this study does not assess and compare clinical effect of different administrations forms but solely evaluate and compare tissue concentrations. Finally, the absence of model-based pharmacokinetic analyses limits the interpretation of the results to the current porcine model and dosing regimen. However, the present study was designed as an exploratory experimental investigation focusing on empirical tissue concentration data obtained through microdialysis, and model-based approaches were therefore beyond its scope. Nonetheless, model-based pharmacokinetic analyses represent an important direction for future research.

## Conclusion

No significant differences in oropharyngeal and frontal sinus tissue T > MIC were found when comparing penicillin bolus with continuous infusion. However, the likelihood of attaining the treatment target of ≥ 50%T > MIC was higher in oropharyngeal tissue following bolus infusion compared to continuous infusion, suggesting that continuous infusion of penicillin may be associated with an increased risk of subtherapeutic concentrations compared to bolus infusion. The findings of this study should be interpreted within the context of the porcine experimental model and the specific dosing regimens applied. Extrapolation to human pharmacokinetics or clinical practice requires further validation through pharmacokinetic modelling and dedicated clinical studies.
